# Pathways to Alzheimer’s Disease: The Intersecting Roles of Clusterin and Apolipoprotein E in Amyloid-β Regulation and Neuronal Health

**DOI:** 10.3390/pathophysiology31040040

**Published:** 2024-10-02

**Authors:** Alexandru Laslo, Laura Laslo, Eliza-Mihaela Arbănași, Alexandru-Andrei Ujlaki-Nagi, Laura Chinezu, Adrian Dumitru Ivănescu, Emil-Marian Arbănași, Roxana Octavia Cărare, Bogdan Andrei Cordoș, Ioana Adriana Popa, Klara Brînzaniuc

**Affiliations:** 1 Department of Urology, George Emil Palade University of Medicine, Pharmacy, Science, and Technology of Targu Mures, 540139 Targu Mures, Romania; alexandru.laslo@gmail.com; 2 Department of Anatomy, George Emil Palade University of Medicine, Pharmacy, Science, and Technology of Targu Mures, 540139 Targu Mures, Romania; adrian.ivanescu@umfst.ro (A.D.I.); klara.brinzaniuc@umfst.ro (K.B.); 3 Doctoral School of Medicine and Pharmacy, George Emil Palade University of Medicine, Pharmacy, Science and Technology of Targu Mures, 540139 Targu Mures, Romania; emilarbanasi1@gmail.com; 4 Faculty of Medicine, George Emil Palade University of Medicine, Pharmacy, Science, and Technology of Targu Mures, 540139 Targu Mures, Romania; laslo.laura2003@yahoo.com (L.L.); bogdan.cordos@umfst.ro (B.A.C.); 5 Regenerative Medicine Laboratory, Centre for Advanced Medical and Pharmaceutical Research (CCAMF), George Emil Palade University of Medicine, Pharmacy, Science, and Technology of Targu Mures, 540139 Targu Mures, Romania; 6 Psychiatry Clinic 2, Mures County Clinical Hospital, 540139 Targu Mures, Romania; 7 Department of Histology, George Emil Palade University of Medicine, Pharmacy, Science, and Technology of Targu Mures, 540139 Targu Mures, Romania; laura.chinezu@umfst.ro; 8 Department of Vascular Surgery, George Emil Palade University of Medicine, Pharmacy, Science and Technology of Targu Mures, 540139 Targu Mures, Romania; 9 Clinic of Vascular Surgery, Mures County Emergency Hospital, 540136 Targu Mures, Romania; 10 Faculty of Medicine, University of Southampton, Southampton SO16 7NS, UK; r.o.carare@soton.ac.uk; 11 Centre for Experimental Medical and Imaging Studies, George Emil Palade University of Medicine, Pharmacy, Science and Technology of Targu Mures, 540139 Targu Mures, Romania; 12 Clinic of Radiology, Mures County Emergency Hospital, 540136 Targu Mures, Romania; ioanaadriana95@gmail.com

**Keywords:** Alzheimer disease, clusterin, apolipoprotein E, amyloid-β, cerebral amyloid angiopathy

## Abstract

One of the hallmarks of Alzheimer’s disease (AD) is the deposition of amyloid-β (Aβ) within the extracellular spaces of the brain as plaques and along the blood vessels in the brain, a condition also known as cerebral amyloid angiopathy (CAA). Clusterin (CLU), or apolipoprotein J (APOJ), is a multifunctional glycoprotein that has a role in many physiological and neurological conditions, including AD. The apolipoprotein E (APOE) is a significant genetic factor in AD, and while the primary physiological role of APOE in the brain and peripheral tissues is to regulate lipid transport, it also participates in various other biological processes, having three basic human forms: APOE2, APOE3, and APOE4. Notably, the APOE4 allele substantially increases the risk of developing late-onset AD. The main purpose of this review is to examine the roles of CLU and APOE in AD pathogenesis in order to acquire a better understanding of AD pathogenesis from which to develop targeted therapeutic approaches.

## 1. Introduction

Alzheimer’s disease (AD) is the most common form of age-related dementia and represents a major health problem in the growing population of elderly people in developed countries [1]. A sizable fraction of neurological illnesses with major implications for the general population is represented by neurodegenerative conditions [2]. The accumulation and aggregation of the peptide Aβ is believed to initiate AD via the amyloid cascade hypothesis [3], whereby abnormal deposition of polymorphous Aβ plaques in the brain parenchyma trigger a pathological cascade, ultimately resulting in neurodegeneration [3,4]. The levels of soluble Aβ are reflective of a balance between its production and elimination from the brain. Whilst familial AD is largely a result of mutations in genes associated with increased production of amyloidogenic peptides cleaved from the Aβ precursor protein (APP), namely, Aβ40 and Aβ42, sporadic forms of AD are traditionally believed to be a consequence of impaired Aβ clearance from the brain. This may be due to altered levels of chaperone proteins, including apolipoprotein E (APOE) and apolipoprotein J (APOJ), also known as clusterin (CLU), that influence Aβ structure, clearance, and toxicity [4].

In addition to the parenchymal accumulation of Aβ plaques, another key element of the neuropathology of AD is the deposition of Aβ within the cerebral blood vessel walls as cerebral amyloid angiopathy (CAA), mainly in cortical arterioles and leptomeningeal arteries [5,6,7,8]. Apart from increasing age, the *ε4* allele of APOE has been recognized as a major risk factor for both AD and CAA [9,10]. Clinical and experimental data demonstrate that the failure of clearance of Aβ from the brain is an essential element of the pathogenesis of AD and a complication after immunization against Aβ [11]. In particular, the failure of clearance of Aβ from the extracellular spaces of the brain along intramural periarterial drainage (IPAD) pathways is a key pathogenic factor in the development of CAA [5,12]. Factors that modify the structure of IPAD pathways for Aβ elimination include the apolipoprotein Eε4 genotype (APOE4), a high-fat diet, and immunization against Aβ [13,14,15,16]. Using atomic force microscopy, recent data reveal that the interactions between the APOE4-Aβ40 complex and the laminin component of vascular basement membranes are significantly weaker than APOE3-Aβ40 complexes, suggesting clearance of Aβ40 via IPAD is less efficient when in complex with APOE4, thus conferring higher risk for AD [17]. Despite APOE being the main lipoprotein of interest with regards to AD risk factors, the emerging role of the lipoprotein CLU (or APOJ) in regulating Aβ aggregation and cytotoxicity in the brain has led to growing interest in this protein as both a mediator and biomarker of AD.

## 2. Clusterin and Alzheimer’s Disease

CLU is a 75-80 KDa disulfide-linked heterodimeric glycoprotein that is constitutively and ubiquitously expressed in most tissues of the body where it functions as an extracellular chaperone and lipoprotein [18]. CLU is abundantly expressed in the CNS and is predominantly synthesized by astrocytes and secreted to the extracellular space [19]. In recent years, emerging association of CLU with AD has made this extracellular chaperone a key protein of interest for improving our understanding of AD pathobiology [20]. Genome-wide association studies of sporadic AD, in which Aβ accumulates both in cortical plaques and CAA, have highlighted the importance of common genetic variations in the gene encoding CLU [21,22].

In late-onset AD, CLU is the third-highest-ranking genetic risk factor, with several AD-associated single nucleotide polymorphisms (SNPs) identified. The effect of such risk variants on CLU expression is complex, with reports of elevated [23,24], decreased [25,26], or unchanged [27,28] levels in brain, plasma, or cerebrospinal fluid (CSF), depending on the study. Despite the inconsistencies relating to CLU gene expression, it is generally accepted that CLU SNPs are significantly associated with increased Aβ deposition [29,30]. Regardless of these variations in gene expression, CLU levels at the protein level are generally increased in patients with AD and correlate with AD disease severity and progression [31]. Increased CLU CSF levels, alongside positive CSF amyloid status, have been associated with a higher atrophy rate of the entorhinal cortex in cognitively normal patients, patients with mild cognitive impairment, and patients with AD, suggesting that CLU may play a role in the earliest stages of the neurodegenerative process of AD [6,32,33]. Furthermore, higher plasma CLU levels are associated with AD prevalence, with higher CLU levels in patients with AD correlating with more severe disease [34]; thus, CLU levels, both in CSF and plasma, may be a useful biomarker of AD. Future research should classify these findings, linking specific SNPs to their effects on CLU mRNA and protein expression.

Investigations using human brain tissue have established that CLU protein levels are elevated in AD, with the highest levels observed in cortical regions with the most abundant deposition of Aβ. However, as a result of the high Aβ levels, the ratio of CLU:Aβ was actually the lowest in these regions. The ability of CLU to reduce aggregation and promote clearance of Aβ is influenced by the relative ratio of CLU to Aβ; for example, elevated levels of CLU in regions of high levels of soluble Aβ result in Aβ-CLU complexes aggregating and depositing in the brain parenchyma. Furthermore, the presence of the APOE4 genotype appears to correlate with CLU levels, which are also associated with the severity of CAA [5,34].

### 2.1. Interactions of CLU with Aβ

The precise role of CLU in modifying the solubility status of Aβ is not fully understood, despite extensive studies that have detected its extracellular colocalization with Aβ deposits [18,35]. As a chaperone protein responsible for clearing misfolded proteins in the interstitial space, including Aβ, CLU has been traditionally viewed as neuroprotective [36]. Some studies have shown that CLU reduces the aggregation and cytotoxicity of Aβ [37,38]. Miners et al. found that the most significant CLU level was found in areas with plaque pathology, roughly corresponding to the Aβ regional distribution. They observed a positive correlation between CLU levels and both insoluble Aβ40 and Aβ42, with CLU levels being considerably higher in patients with AD compared to controls. The amount of soluble CLU increased considerably with the degree of CAA and with the highest level of CLU in homozygotes of APOE4 [3]. However, further research is needed to determine whether other metabolic or molecular markers are associated with APOE4 homozygosity and if this genotype impacts other neurodegenerative or pathophysiological conditions influencing neurodegeneration.

Using highly sensitive single-molecule fluorescence methods, it has been shown specifically that the oligomeric soluble forms of Aβ40 interact with CLU to form stable complexes [39,40]. However, synthesizing pure recombinant CLU is challenging because the experimental conditions often lead to the simultaneous formation of misfolded proteins in the media, attaching to the CLU and interfering with its biological properties [40,41]. This misfolding makes it difficult to obtain CLU in its pure form under laboratory conditions, though it is possible to synthesize recombinant CLU in its physiological form, which more accurately reflects the naturally occurring CLU in the body [42]. CLU was shown to prevent the toxicity of Aβ42 oligomers in glial cells and neurons, as well as to improve memory in a rodent model [43]. However, there are studies that have contradicting results, with CLU leading to an increase in oxidative stress and neurotoxicity in rodent brain slices [2,44,45]. These effects may be related to higher molar ratios of amyloid relative to CLU, which result in an increase in aggregates of amyloid. Reactive oxygen and nitrogen species concentrations increase in tandem with a decrease in endogenous antioxidant production, thus indicating an imbalance between the pro-oxidant species and the natural antioxidant systems, leading, in turn, to a state of oxidative stress [2].

### 2.2. Role of CLU in Aβ Clearance from the Brain

Effective and efficient clearance of Aβ from the brain is key to preventing its pathological accumulation, a key factor underlying amyloidosis and AD risk. The major pathways responsible for Aβ elimination are proteolytic degradation by extracellular proteases including neprilysin and the insulin-degrading enzyme [46,47]; clearance across the blood–brain barrier (BBB) via endocytic membrane receptors, including low-density-lipoprotein-related protein 1 (LRP-1) and 2 (LRP-2) [47]; and the perivascular drainage via the basement membrane of cerebral capillaries and arteries to the cervical lymph nodes via IPAD. Each pathway interacts with others, creating a complex network that regulates Aβ clearance, with their relative importance potentially varying based on Aβ species and brain regions.

Aβ40, due to its more soluble nature, is predominantly found in the vasculature, whilst the more fibrillogenic Aβ42 is predominantly deposited as plaques in the brain parenchyma. The ratio of Aβ40 to Aβ42 is therefore indicative of the location of Aβ deposition in the brain, with higher ratios predisposing vascular deposition and CAA [48]. This suggests that pathways involved in Aβ40 clearance are more active in the vascular regions, whereas those involved in Aβ42 clearance are more active in preventing parenchymal deposition. Early in vitro studies demonstrated that CLU prevents Aβ aggregation [48] and that transport of Aβ42 through the BBB via the endocytic receptor (LRP2) is significantly enhanced when complexed with CLU [49]. CLU appears to be sequestered with Aβ species in sporadic CAA as well as in the white matter abnormalities in cerebral autosomal dominant arteriopathy with subcortical infarcts and leukoencephalopathy (CADASIL) [50,51,52]. Although the predominant species of Aβ in CAA is Aβ40, the progressive failure of IPAD leads to entrapment and accumulation of Aβ42 in the walls of blood vessels, aligned with a significant positive correlation between CLU concentration and regional levels of insoluble Aβ42 in human brains. These findings are a possible consequence of entrapment of the Aβ-CLU complex in the IPAD pathways, or may represent a compensatory upregulation of CLU to clear the excess Aβ42 that cannot be eliminated normally [53]. The interconnections and hierarchy of clearance mechanisms, as well as the strength and localisation of CLU and Aβ species, are expected to impact the overall result of Aβ deposition and AD development.

### 2.3. Cellular Risk Factors Associated with CLU Protein in Alzheimer’s Disease: Implications for Lipid Metabolism, Homeostasis, and Neuronal Apoptosis

*CLU,* encoded by the APOJ gene, is the second-most-important apolipoprotein in the neurological system [33,54] and influences the transport of phospholipids and cholesterol. APOJ is a key cholesterol-carrying lipoprotein within brain tissue [55,56,57,58]. The significance of lipoprotein-mediated cholesterol transport in the brain is highlighted by the role cholesterol plays in the development of AD. This pathway affects systems relevant to Aβ and lipid metabolism [59].

High CLU levels have been correlated with atherosclerosis, and CLU appears to be involved in the release of cholesterol from lipid-laden macrophage (LLM) [33,55]. Recent research has linked APOJ polymorphisms to carotid intima-media thickness, as well as the lipid content. These findings suggest that genetic differences in CLU may indirectly impact AD sensitivity by increasing the chances of cerebrovascular disorders, which might therefore lead to neurodegenerative diseases. Cholesterol transport in the brain is particularly significant given the role that cholesterol plays in AD development, lipids being essential for myelin formation and neuronal function [57]. Since lipids are insoluble in water, they have to be dissolved and carried as lipoprotein fragments to span non-adjacent cells [57,58]. Further investigations are required to determine if the presence of CLU polymorphisms directly modifies the metabolic alterations that are seen along the course of disease development, and if it has an indirect effect on brain lipid metabolism via amyloid activity or possible pathways in the cerebral circulation [59].

Copper homeostasis is another pathway implicated in AD development, and disruptions in copper homeostasis represent one of the mechanisms implicated in AD pathogenesis. It has been demonstrated that oxidative stress and ATP7B modifications affect the interaction between copper ATPases and CLU. This indicates that CLU-linked ATP-7A and CLU-linked ATP-7B may be broken down in part by peroxidation brought on by reduced copper levels. The two SNPs, rs_732774 and rs_1061472, found in ATP7B, occur in areas of the protein that encode essential transmembrane and transduction domains. Those modifications have been linked to an elevated likelihood of developing AD [60].

The activation of glial cells throughout early stages of AD is thought to be a pathological reaction against the buildup of Aβ [61]. This response is aligned with the trajectory of cognitive decline in patients with AD [62]. Reactive astrocytes/astrogliosis can be detected by measuring cerebrospinal fluid biomarkers such as glial fibrillary acidic protein (GFAP), chitinase-3-like protein 1 (YKL-40), or S100B. These modifications are linked to a variety of physiological processes in AD, including tau pathology, glucose metabolism, and inflammation [63].

Nuclear CLU (nCLU) and secreted CLU (sCLU) differ in their domain structures and functions; nCLU promotes apoptosis, while sCLU helps cells survive. These isoforms have influence on a variety of biological activities, including cell cycle progression, apoptosis, and DNA repair [64,65]. Studies have shown that overexpression on full-length APOJ mRNA can result in a non-physiological synthesis of nCLU, which could function as a pro-death signaling pathway, influencing cell growth and survival [66,67]. Prolonged tumor cell survival results from removing the nCLU and increasing the sCLU expression, and research supports the idea that blocking sCLU is needed for triggering cell death [66,67].

CLU also participates in DNA repair pathways, including the nonhomologous end-joining route, with nCLU forming a trimeric protein structure with Ku-80 when it interacts with Ku-70. The Ku autoantigen represents a heterodimer containing 70 kDa (Ku70) and 80 kDa (Ku80) proteins that binds nuclear DNA end-to-end. Once attached to the DNA lesions, this complex influences apoptosis, cell cycle arrest, carcinogenesis, and DNA repair, while also signaling damaging stress responses. Ku70 and Ku80 could also be involved in more fundamental biological functions, such telomerase function(s) [68]. The increased expression of nCLU reduces the ability of Ku-80/Ku-70 to bind DNA in cellular extracts, with CLU regulating cell cycle growth. Increased levels of sCLU are related to G1 phase delay in a variety of cell types. These abnormalities have been related to the onset of AD [69].

Taken together, the results summarized above indicate that clusterin is protective against apoptosis generated by different stimuli, consistent with an increasing amount of data indicating that higher clusterin expression in tumor cells is protective against the death of cells via apoptosis [69,70]. Although clusterin appears to originate from modifications after translation of a single mRNA transcript, it may arise in multiple molecular configurations. Whenever clusterin is upregulated in response to different apoptotic cues, it indicates an adaptive cell-survival process [69].

## 3. APOE and Alzheimer’s Disease

### 3.1. APOE–Isoforms and Structure

**Figure 1:**
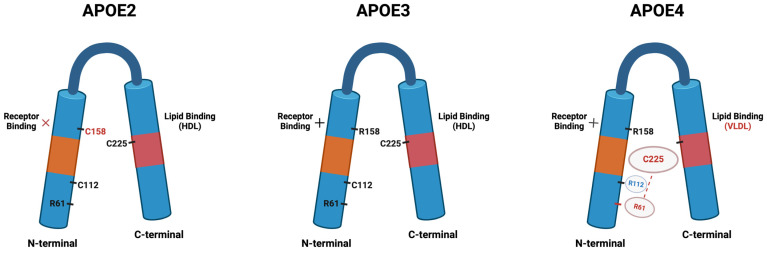
The morphology of APOE isoforms. APOE is a soluble protein, possessing N-terminal and C-terminal domains that are connected by a central hinge region. The N-terminal domain contains the receptor-binding domain (orange), and the C-terminal domain contains the lipid-binding region (red). The isoforms are different from each other at amino acid positions 112 and 158. Position 158 (C158) in APOE2 hosts cysteine, leading to deficient receptor binding; position 112 (R112) in APOE4 hosts arginine. This changes the conformation of the domain as R61 is exposed and interacts with C255 at the C-terminal domain (shown with the red dotted line). This phenomenon of “domain interaction” represents the biophysical basis for differences in the properties and function of APOE4 compared to the other isoforms; for example, the preference for very-low-density lipoproteins over high-density lipoproteins. In APOE2 and APOE3, which possess C112 rather than R112, the R61 is hidden and there is no such interaction at this domain. Image produced with BioRender, recreated from Celia G. Fernandez et al. [72].

Apolipoprotein E (APOE) is expressed in six major genotypes, three of which are homozygous: APOE2/2, APOE3/3, and APOE4/4; and three of which are heterozygous: APOE2/3, APOE2/4, and APOE3/4, which are distinguished by the amino acid differences in the positions 112 and 158 [71,72] (shown in Figure 1).

APOE has three core domains: an N-terminal domain, a C-terminal domain, and a connecting adaptable hinged domain. The N-terminal and the C-terminal domains are important for the binding of receptors and lipids, respectively. The differences in the amino acid structures between the APOE isoforms occur from various protein foldings, influencing their lipid- and receptor-binding characteristics [73]. On the other hand, the APOE gene in mice (m-APOE) is located on the seventh chromosome and has only one isoform. The promoting areas —the cingulate cortex, the molecular layer of dentate gyrus, and the hippocampus in human and mouse APOE—possess under 40% similarities [74]. There are notable functional variations between human and mouse APOE protein in the AD-related functions, including Aβ clearance, neurological inflammation, and synapse structure [75,76].

Due to the specific functional characteristics and polymorphisms of APOE, the frequency distribution of APOE alleles in individuals must be evaluated. The overall frequencies of the APOE alleles are as follows: E2 with 7%; E4 with 14%; and E3 with 79% [77,78]. Recent studies have clarified the frequency of the different APOE alleles. Variations in APOE alleles have been linked to differences in susceptibility to neurodegenerative diseases, the APOE4 allele is strongly associated with an increased risk of AD, while the APOE2 allele is associated with a decreased risk [79,80,81].

The TOMM40 gene, located near the APOE gene, is involved in mitochondrial function and has been shown to interact with APOE in terms of gene regulation, and variants in the TOMM40 gene can affect APOE expression levels and contribute to differences in AD risk [78].

To summarize, the variations between APOE isoforms are due to amino acid alterations that impact protein folding and function. The promoter regions of human and mouse APOE are quite different, which influences gene regulation. The frequency of APOE alleles is associated with neurodegenerative risk, and interactions with adjacent genes such as TOMM40 influence APOE’s function in disease processes.

### 3.2. APOE–Secretion and Production

Both the production and elimination of the protein(s) encoded by the APOE gene have cellular and tissue particularities and can both be increased by a variety of transcription factors, presence of hormones, cytokines, and lipids [82]. Oligodendrocytes, microglia, the choroid plexus, and neurons under stress may also synthesize ApoE but to a lesser extent [83,84,85,86]. ApoE is produced in the endoplasmic reticulum, post-translationally changed in the Golgi apparatus, transported to the membrane of the plasma cell, and ultimately secreted [87]. The importance of protein glycosylation in AD has been studied intensively, concluding that astrocyte-derived ApoE show greater levels of sialylation, as well as glycosylation [87,88,89,90], and demonstrating the presence of tissue-specific ApoE glycoforms [91].

**Figure 2:**
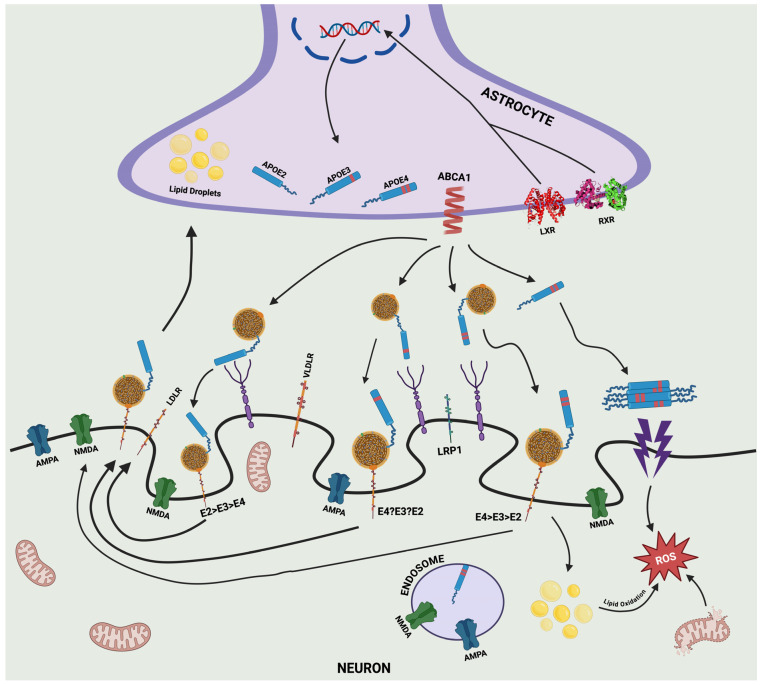
The roles of APOE isoforms (APOE2/3/4) in the interactions between astrocytes and neurons. Activation of liver X receptor (LXR) and retinoid X receptor (RXR) regulate the expression of isoforms of APOE in astrocytes. ATP-binding cassette transporter (ABCA1) is responsible for the lipidation of the protein ApoE encoded by APOE and its secretion to the extracellular space. ApoE in the extracellular space binds to several neuronal lipid receptors, including very-low-density lipoprotein receptor (VLDLR), low-density lipoprotein receptor (LDLR), low-density lipoprotein receptor-related protein 1 (LRP1), and heparan sulfate proteoglycans (HSPGs). The lipidation status of APOE isoforms promotes individual receptor affinities. The apoE4 isoform has a low-lipidation status, forms non-lipidated apoE4 aggregates, and has a poor lipid turnover, leading, in turn, to neuronal lipid droplet accumulation. This results in damage to the mitochondria and production of reactive oxygen species (ROS). ApoE4 promotes the formation of endosomes and the degradation of synaptic receptors such as AMPA or NMDA and the consequent impaired synaptic function. Image produced with BioRender, recreated from R. Fernandez-Calle et al. [102].

DNA-binding factors like the liver X receptor (LXR) and the retinoid X receptor (RXR) regulate APOE production and elimination. LXR also affects the immune system response during the inflammatory events [92,93,94]. The expression of APOE and the efflux of cholesterol from astrocytes can be stimulated by brain-derived neurotrophic factor (BDNF) via the activation of LXR [94]. LXR and RXR are both involved in the transcriptional regulation of APOE [95]. RXR is mostly present in hepatocytes. It is connected with the recovery mechanisms after stroke [96,97,98] and it regulates the gene translation of the ATP binding cassette transporter A1 (ABCA1) [99], which is an essential transporter that promotes the flow of cholesterol via lipoproteins, required for the development of high-density lipoproteins (HDL) [100,101] (Figure 2).

APOE4 inhibits the membrane recycling of ABCA1 [102]. The brain is the most cholesterol-rich organ, containing approximately 20% of the total cholesterol in the body; therefore, APOE has an important function in lipid transport and cholesterol homeostasis through transporting cholesterol to neurons and eliminating excessive cholesterol [103,104]. APOE’s function in lipid transport and cholesterol homeostasis in the brain is important for maintaining cellular membrane integrity, neuronal plasticity, signal transmission, and proteostasis. Disruptions in APOE production, post-translational modifications, and regulation by DNA-binding factors and kinases can significantly impact AD progression and neuropathology [105,106,107].

### 3.3. Distribution of APOE in the Blood, Brain, and Cerebrospinal Fluid

In the past few years, there has been increasing interest in monitoring APOE levels in biological fluids. APOE, a major lipoprotein in the CSF, largely interacts with HDLs and promotes cholesterol transport throughout the CNS [108]. The specific isoforms of APOE affect HDL particle transport, cognitive function, with direct correlations to the onset of neurodegenerative disorders [109,110]. Studies on APOE levels in the brains of the humanized APOE-knock-in mice have revealed an isoform-related decrease in APOE levels, with levels of APOE higher in the APOE2 compared toAPOE3 and higher in the APOE3 compared to APOE4 [111,112]. Newly generated ApoE4 degrades faster and has a shorter half-life than APOE3 [113].

The cerebellum showed the highest expression of APOE, whereas the hippocampus showed the lowest levels. Furthermore, APOE levels in transgenic mice expressing human APOE2, APOE3, and APOE4 revealed that APOE2 values were 16 times higher compared to the other two isoforms. These findings are in contrast with those in the non-obese diabetic (NOD), in which the hippocampus had the greatest amount of endogenous APOE and the thalamus had the lowest [114]. Further studies based on the APOE-targeted replacement mice showed that APOE4 had lower CSF APOE levels compared to mice expressing the other isoforms [115,116].

There are two distinct sources of APOE: peripheral APOE, which is primarily generated by hepatocytes; and CNS APOE, which is predominantly produced by astrocytes [117,118]. Investigations on APOE-knock-in mice have demonstrated that removing the hepatic APOE alleles does not change the cerebral APOE levels, although it does modify plasma lipid composition and lowers the APOE concentrations in plasma. Restoring the levels of APOE in the plasma of APOE-knock-out-mice resulted in normalizing plasma lipids, and although this did not prevent synapse loss, it did improve the memory and learning deficiencies [106,119]. This indicates that the APOE present in CNS or plasma have distinct effects on brain function [3,120].

Investigations on APOE levels in the human CSF and plasma have showed results that do not correspond to those reported in mice. For example, the APOE genotype has little effect on the APOE levels in CSF [120,121]. However, neither the total CSF APOE concentration nor any specific isoforms have been associated with the Aβ accumulation or the severity of dementia in patients with AD [122].

Various genetic and environmental variables influencing neurodegeneration and neuroinflammation also contribute to differences in memory function among age groups. For example, middle-aged APOE4 carriers experience slower memory deterioration [123]. Still, it remains unclear as to how the overall APOE concentration or its particular isoforms relate to Aβ, phosphorylated protein tau levels, the degree of severity of dementia, or other biological parameters.

## 4. Conclusions

The rising CLU levels observed in a variety of neurodegenerative diseases are aligned with a toxic cerebral protein aggregation. APOE isoforms are biologically linked to neurodegenerative disorders, with APOE4 representing the highest risk factor of Alzheimer’s disease. CLU appears to have roles in neuroprotection as well as neurodegeneration. Understanding the exact roles of CLU is mostly difficult due to its complicated biology, which results in different physiological activities. The development of innovative efficient AD treatment alternatives may harness the neuroprotective roles of CLU and will need to consider the influence and effects of APOE isoforms.

## References

[1] Alzheimer’s Association (2014). 2014 Alzheimer’s Disease Facts and Figures. Alzheimers Dement. J. Alzheimers Assoc..

[2] Olufunmilayo E.O., Gerke-Duncan M.B., Holsinger R.M.D. (2023). Oxidative Stress and Antioxidants in Neurodegenerative Disorders. Antioxidants.

[3] Miners J.S., Clarke P., Love S. (2017). Clusterin Levels Are Increased in Alzheimer’s Disease and Influence the Regional Distribution of Aβ. Brain Pathol. Zurich Switz..

[4] Barage S.H., Sonawane K.D. (2015). Amyloid Cascade Hypothesis: Pathogenesis and Therapeutic Strategies in Alzheimer’s Disease. Neuropeptides.

[5] Vinters H.V. (1987). Cerebral Amyloid Angiopathy. A Critical Review. Stroke.

[6] Jellinger K.A. (2002). Alzheimer Disease and Cerebrovascular Pathology: An Update. J. Neural Transm. Vienna Austria 1996.

[7] Roher A.E., Kuo Y.-M., Esh C., Knebel C., Weiss N., Kalback W., Luehrs D.C., Childress J.L., Beach T.G., Weller R.O. (2003). Cortical and Leptomeningeal Cerebrovascular Amyloid and White Matter Pathology in Alzheimer’s Disease. Mol. Med. Camb. Mass.

[8] Revesz T., Holton J.L., Lashley T., Plant G., Rostagno A., Ghiso J., Frangione B. (2002). Sporadic and Familial Cerebral Amyloid Angiopathies. Brain Pathol. Zurich Switz..

[9] Corder E.H., Saunders A.M., Strittmatter W.J., Schmechel D.E., Gaskell P.C., Small G.W., Roses A.D., Haines J.L., Pericak-Vance M.A. (1993). Gene Dose of Apolipoprotein E Type 4 Allele and the Risk of Alzheimer’s Disease in Late Onset Families. Science.

[10] Greenberg S.M., Briggs M.E., Hyman B.T., Kokoris G.J., Takis C., Kanter D.S., Kase C.S., Pessin M.S. (1996). Apolipoprotein E Epsilon 4 Is Associated with the Presence and Earlier Onset of Hemorrhage in Cerebral Amyloid Angiopathy. Stroke.

[11] Tarasoff-Conway J.M., Carare R.O., Osorio R.S., Glodzik L., Butler T., Fieremans E., Axel L., Rusinek H., Nicholson C., Zlokovic B.V. (2015). Clearance Systems in the Brain-Implications for Alzheimer Disease. Nat. Rev. Neurol..

[12] Weller R.O., Subash M., Preston S.D., Mazanti I., Carare R.O. (2008). Perivascular Drainage of Amyloid-Beta Peptides from the Brain and Its Failure in Cerebral Amyloid Angiopathy and Alzheimer’s Disease. Brain Pathol. Zurich Switz..

[13] Hawkes C.A., Härtig W., Kacza J., Schliebs R., Weller R.O., Nicoll J.A., Carare R.O. (2011). Perivascular Drainage of Solutes Is Impaired in the Ageing Mouse Brain and in the Presence of Cerebral Amyloid Angiopathy. Acta Neuropathol..

[14] Hawkes C.A., Sullivan P.M., Hands S., Weller R.O., Nicoll J.A.R., Carare R.O. (2012). Disruption of Arterial Perivascular Drainage of Amyloid-β from the Brains of Mice Expressing the Human APOE Ε4 Allele. PLoS ONE.

[15] Hawkes C.A., Gentleman S.M., Nicoll J.A., Carare R.O. (2015). Prenatal High-Fat Diet Alters the Cerebrovasculature and Clearance of β-Amyloid in Adult Offspring. J. Pathol..

[16] Carare R.O., Teeling J.L., Hawkes C.A., Püntener U., Weller R.O., Nicoll J.A., Perry V.H. (2013). Immune Complex Formation Impairs the Elimination of Solutes from the Brain: Implications for Immunotherapy in Alzheimer’s Disease. Acta Neuropathol. Commun..

[17] Zekonyte J., Sakai K., Nicoll J.A.R., Weller R.O., Carare R.O. (2016). Quantification of Molecular Interactions between ApoE, Amyloid-Beta (Aβ) and Laminin: Relevance to Accumulation of Aβ in Alzheimer’s Disease. Biochim. Biophys. Acta.

[18] Foster E.M., Dangla-Valls A., Lovestone S., Ribe E.M., Buckley N.J. (2019). Clusterin in Alzheimer’s Disease: Mechanisms, Genetics, and Lessons From Other Pathologies. Front. Neurosci..

[19] Nuutinen T., Suuronen T., Kauppinen A., Salminen A. (2009). Clusterin: A Forgotten Player in Alzheimer’s Disease. Brain Res. Rev..

[20] Yuste-Checa P., Bracher A., Hartl F.U. (2022). The Chaperone Clusterin in Neurodegeneration-Friend or Foe?. BioEssays News Rev. Mol. Cell. Dev. Biol..

[21] Lambert J.-C., Heath S., Even G., Campion D., Sleegers K., Hiltunen M., Combarros O., Zelenika D., Bullido M.J., Tavernier B. (2009). Genome-Wide Association Study Identifies Variants at CLU and CR1 Associated with Alzheimer’s Disease. Nat. Genet..

[22] Jansen I.E., Savage J.E., Watanabe K., Bryois J., Williams D.M., Steinberg S., Sealock J., Karlsson I.K., Hägg S., Athanasiu L. (2019). Genome-Wide Meta-Analysis Identifies New Loci and Functional Pathways Influencing Alzheimer’s Disease Risk. Nat. Genet..

[23] Szymanski M., Wang R., Bassett S.S., Avramopoulos D. (2011). Alzheimer’s Risk Variants in the Clusterin Gene Are Associated with Alternative Splicing. Transl. Psychiatry.

[24] Padhy B., Nanda G.G., Chowdhury M., Padhi D., Rao A., Alone D.P. (2014). Role of an Extracellular Chaperone, Clusterin in the Pathogenesis of Pseudoexfoliation Syndrome and Pseudoexfoliation Glaucoma. Exp. Eye Res..

[25] Xing Y.-Y., Yu J.-T., Cui W.-Z., Zhong X.-L., Wu Z.-C., Zhang Q., Tan L. (2012). Blood Clusterin Levels, Rs9331888 Polymorphism, and the Risk of Alzheimer’s Disease. J. Alzheimers Dis. JAD.

[26] Allen M., Zou F., Chai H.S., Younkin C.S., Crook J., Pankratz V.S., Carrasquillo M.M., Rowley C.N., Nair A.A., Middha S. (2012). Novel Late-Onset Alzheimer Disease Loci Variants Associate with Brain Gene Expression. Neurology.

[27] Karch C.M., Jeng A.T., Nowotny P., Cady J., Cruchaga C., Goate A.M. (2012). Expression of Novel Alzheimer’s Disease Risk Genes in Control and Alzheimer’s Disease Brains. PLoS ONE.

[28] Deming Y., Xia J., Cai Y., Lord J., Holmans P., Bertelsen S., Holtzman D., Morris J.C., Bales K., Pickering E.H. (2016). A Potential Endophenotype for Alzheimer’s Disease: Cerebrospinal Fluid Clusterin. Neurobiol. Aging.

[29] Tan L., Wang H.-F., Tan M.-S., Tan C.-C., Zhu X.-C., Miao D., Yu W.-J., Jiang T., Tan L., Yu J.-T. (2016). Effect of CLU Genetic Variants on Cerebrospinal Fluid and Neuroimaging Markers in Healthy, Mild Cognitive Impairment and Alzheimer’s Disease Cohorts. Sci. Rep..

[30] DeMattos R.B., Cirrito J.R., Parsadanian M., May P.C., O’Dell M.A., Taylor J.W., Harmony J.A.K., Aronow B.J., Bales K.R., Paul S.M. (2004). ApoE and Clusterin Cooperatively Suppress Abeta Levels and Deposition: Evidence That ApoE Regulates Extracellular Abeta Metabolism in Vivo. Neuron.

[31] Lidström A.M., Bogdanovic N., Hesse C., Volkman I., Davidsson P., Blennow K. (1998). Clusterin (Apolipoprotein J) Protein Levels Are Increased in Hippocampus and in Frontal Cortex in Alzheimer’s Disease. Exp. Neurol..

[32] Desikan R.S., Thompson W.K., Holland D., Hess C.P., Brewer J.B., Zetterberg H., Blennow K., Andreassen O.A., McEvoy L.K., Hyman B.T. (2014). The Role of Clusterin in Amyloid-β Associated Neurodegeneration. JAMA Neurol..

[33] Thambisetty M., Simmons A., Velayudhan L., Hye A., Campbell J., Zhang Y., Wahlund L.-O., Westman E., Kinsey A., Güntert A. (2010). Association of Plasma Clusterin Concentration with Severity, Pathology, and Progression in Alzheimer Disease. Arch. Gen. Psychiatry.

[34] Schrijvers E.M.C., Koudstaal P.J., Hofman A., Breteler M.M.B. (2011). Plasma Clusterin and the Risk of Alzheimer Disease. JAMA.

[35] Fandos N., Pérez-Grijalba V., Pesini P., Olmos S., Bossa M., Villemagne V.L., Doecke J., Fowler C., Masters C.L., Sarasa M. (2017). Plasma amyloid β 42/40 ratios as biomarkers for amyloid β cerebral deposition in cognitively normal individuals. Alzheimers Dement..

[36] Giannakopoulos P., Kövari E., French L.E., Viard I., Hof P.R., Bouras C. (1998). Possible Neuroprotective Role of Clusterin in Alzheimer’s Disease: A Quantitative Immunocytochemical Study. Acta Neuropathol..

[37] Narayan P., Holmström K.M., Kim D.-H., Whitcomb D.J., Wilson M.R., St George-Hyslop P., Wood N.W., Dobson C.M., Cho K., Abramov A.Y. (2014). Rare Individual Amyloid-β Oligomers Act on Astrocytes to Initiate Neuronal Damage. Biochemistry.

[38] DeMattos R.B., O’dell M.A., Parsadanian M., Taylor J.W., Harmony J.A.K., Bales K.R., Paul S.M., Aronow B.J., Holtzman D.M. (2002). Clusterin Promotes Amyloid Plaque Formation and Is Critical for Neuritic Toxicity in a Mouse Model of Alzheimer’s Disease. Proc. Natl. Acad. Sci. USA.

[39] Narayan P., Orte A., Clarke R.W., Bolognesi B., Hook S., Ganzinger K.A., Meehan S., Wilson M.R., Dobson C.M., Klenerman D. (2011). The Extracellular Chaperone Clusterin Sequesters Oligomeric Forms of the Amyloid-β(1-40) Peptide. Nat. Struct. Mol. Biol..

[40] Lakins J.N., Poon S., Easterbrook-Smith S.B., Carver J.A., Tenniswood M.P.R., Wilson M.R. (2002). Evidence That Clusterin Has Discrete Chaperone and Ligand Binding Sites. Biochemistry.

[41] Poon S., Treweek T.M., Wilson M.R., Easterbrook-Smith S.B., Carver J.A. (2002). Clusterin Is an Extracellular Chaperone That Specifically Interacts with Slowly Aggregating Proteins on Their Off-Folding Pathway. FEBS Lett..

[42] Dabbs R.A., Wilson M.R. (2014). Expression and Purification of Chaperone-Active Recombinant Clusterin. PLoS ONE.

[43] Cascella R., Conti S., Tatini F., Evangelisti E., Scartabelli T., Casamenti F., Wilson M.R., Chiti F., Cecchi C. (2013). Extracellular Chaperones Prevent Aβ42-Induced Toxicity in Rat Brains. Biochim. Biophys. Acta.

[44] Oda T., Wals P., Osterburg H.H., Johnson S.A., Pasinetti G.M., Morgan T.E., Rozovsky I., Stine W.B., Snyder S.W., Holzman T.F. (1995). Clusterin (apoJ) Alters the Aggregation of Amyloid Beta-Peptide (A Beta 1-42) and Forms Slowly Sedimenting A Beta Complexes That Cause Oxidative Stress. Exp. Neurol..

[45] Lambert M.P., Barlow A.K., Chromy B.A., Edwards C., Freed R., Liosatos M., Morgan T.E., Rozovsky I., Trommer B., Viola K.L. (1998). Diffusible, Nonfibrillar Ligands Derived from Abeta1-42 Are Potent Central Nervous System Neurotoxins. Proc. Natl. Acad. Sci. USA.

[46] Jha N.K., Jha S.K., Kumar D., Kejriwal N., Sharma R., Ambasta R.K., Kumar P. (2015). Impact of Insulin Degrading Enzyme and Neprilysin in Alzheimer’s Disease Biology: Characterization of Putative Cognates for Therapeutic Applications. J. Alzheimers Dis. JAD.

[47] Deane R., Bell R.D., Sagare A., Zlokovic B.V. (2009). Clearance of Amyloid-Beta Peptide across the Blood-Brain Barrier: Implication for Therapies in Alzheimer’s Disease. CNS Neurol. Disord. Drug Targets.

[48] Matsubara E., Soto C., Governale S., Frangione B., Ghiso J. (1996). Apolipoprotein J and Alzheimer’s Amyloid Beta Solubility. Biochem. J..

[49] Bell R.D., Sagare A.P., Friedman A.E., Bedi G.S., Holtzman D.M., Deane R., Zlokovic B.V. (2007). Transport Pathways for Clearance of Human Alzheimer’s Amyloid Beta-Peptide and Apolipoproteins E and J in the Mouse Central Nervous System. J. Cereb. Blood Flow Metab. Off. J. Int. Soc. Cereb. Blood Flow Metab..

[50] Calero M., Rostagno A., Matsubara E., Zlokovic B., Frangione B., Ghiso J. (2000). Apolipoprotein J (Clusterin) and Alzheimer’s Disease. Microsc. Res. Tech..

[51] Howlett D.R., Hortobágyi T., Francis P.T. (2013). Clusterin Associates Specifically with Aβ40 in Alzheimer’s Disease Brain Tissue. Brain Pathol..

[52] Craggs L., Taylor J., Slade J.Y., Chen A., Hagel C., Kuhlenbaeumer G., Borjesson-Hanson A., Viitanen M., Kalimo H., Deramecourt V. (2016). Clusterin/Apolipoprotein J Immunoreactivity Is Associated with White Matter Damage in Cerebral Small Vessel Diseases. Neuropathol. Appl. Neurobiol..

[53] Manousopoulou A., Gatherer M., Smith C., Nicoll J.A.R., Woelk C.H., Johnson M., Kalaria R., Attems J., Garbis S.D., Carare R.O. (2017). Systems Proteomic Analysis Reveals That Clusterin and Tissue Inhibitor of Metalloproteinases 3 Increase in Leptomeningeal Arteries Affected by Cerebral Amyloid Angiopathy. Neuropathol. Appl. Neurobiol..

[54] Raulin A.C., Doss S.V., Trottier Z.A., Ikezu T.C., Bu G., Liu C.C. (2022). ApoE in Alzheimer’s disease: Pathophysiology and therapeutic strategies. Mol. Neurodegener..

[55] Ishikawa Y., Akasaka Y., Ishii T., Komiyama K., Masuda S., Asuwa N., Choi-Miura N.H., Tomita M. (1998). Distribution and Synthesis of Apolipoprotein J in the Atherosclerotic Aorta. Arterioscler. Thromb. Vasc. Biol..

[56] Squitti R., Polimanti R., Bucossi S., Ventriglia M., Mariani S., Manfellotto D., Vernieri F., Cassetta E., Ursini F., Rossini P.M. (2013). Linkage Disequilibrium and Haplotype Analysis of the ATP7B Gene in Alzheimer’s Disease. Rejuvenation Res..

[57] Elliott D.A., Weickert C.S., Garner B. (2010). Apolipoproteins in the Brain: Implications for Neurological and Psychiatric Disorders. Clin. Lipidol..

[58] Lee K.-B., Jeon J.-H., Choi I., Kwon O.-Y., Yu K., You K.-H. (2008). Clusterin, a Novel Modulator of TGF-Beta Signaling, Is Involved in Smad2/3 Stability. Biochem. Biophys. Res. Commun..

[59] Zhou Y., Wang Y., Tischfield M., Williams J., Smallwood P.M., Rattner A., Taketo M.M., Nathans J. (2014). Canonical WNT Signaling Components in Vascular Development and Barrier Formation. J. Clin. Investig..

[60] Materia S., Cater M.A., Klomp L.W.J., Mercer J.F.B., La Fontaine S. (2011). Clusterin (Apolipoprotein J), a Molecular Chaperone That Facilitates Degradation of the Copper-ATPases ATP7A and ATP7B. J. Biol. Chem..

[61] Janelidze S., Mattsson N., Stomrud E., Lindberg O., Palmqvist S., Zetterberg H., Blennow K., Hansson O. (2018). CSF Biomarkers of Neuroinflammation and Cerebrovascular Dysfunction in Early Alzheimer Disease. Neurology.

[62] Ewers M., Franzmeier N., Suárez-Calvet M., Morenas-Rodriguez E., Caballero M.A.A., Kleinberger G., Piccio L., Cruchaga C., Deming Y., Dichgans M. (2019). Increased Soluble TREM2 in Cerebrospinal Fluid Is Associated with Reduced Cognitive and Clinical Decline in Alzheimer’s Disease. Sci. Transl. Med..

[63] Bellaver B., Ferrari-Souza J.P., Uglione da Ros L., Carter S.F., Rodriguez-Vieitez E., Nordberg A., Pellerin L., Rosa-Neto P., Leffa D.T., Zimmer E.R. (2021). Astrocyte Biomarkers in Alzheimer Disease: A Systematic Review and Meta-Analysis. Neurology.

[64] Moretti R.M., Montagnani Marelli M., Mai S., Cariboni A., Scaltriti M., Bettuzzi S., Limonta P. (2007). Clusterin Isoforms Differentially Affect Growth and Motility of Prostate Cells: Possible Implications in Prostate Tumorigenesis. Cancer Res..

[65] Rizzi F., Coletta M., Bettuzzi S. (2009). Chapter 2: Clusterin (CLU): From One Gene and Two Transcripts to Many Proteins. Adv. Cancer Res..

[66] Xie Z., Harris-White M.E., Wals P.A., Frautschy S.A., Finch C.E., Morgan T.E. (2005). Apolipoprotein J (Clusterin) Activates Rodent Microglia in Vivo and in Vitro. J. Neurochem..

[67] Cunin P., Beauvillain C., Miot C., Augusto J.-F., Preisser L., Blanchard S., Pignon P., Scotet M., Garo E., Fremaux I. (2016). Clusterin Facilitates Apoptotic Cell Clearance and Prevents Apoptotic Cell-Induced Autoimmune Responses. Cell Death Dis..

[68] Yang C.R., Leskov K., Hosley-Eberlein K., Criswell T., Pink J.J., Kinsella T.J., Boothman D.A. (2000). Nuclear Clusterin/XIP8, an x-Ray-Induced Ku70-Binding Protein That Signals Cell Death. Proc. Natl. Acad. Sci. USA.

[69] Zellweger T., Kiyama S., Chi K., Miyake H., Adomat H., Skov K., Gleave M.E. (2003). Overexpression of the Cytoprotective Protein Clusterin Decreases Radiosensitivity in the Human LNCaP Prostate Tumour Model. BJU Int..

[70] Zellweger T., Miyake H., Cooper S., Chi K., Conklin B.S., Monia B.P., Gleave M.E. (2001). Antitumor Activity of Antisense Clusterin Oligonucleotides Is Improved in Vitro and in Vivo by Incorporation of 2’-O-(2-Methoxy)Ethyl Chemistry. J. Pharmacol. Exp. Ther..

[71] Chen Y., Strickland M.R., Soranno A., Holtzman D.M. (2021). Apolipoprotein E: Structural Insights and Links to Alzheimer Disease Pathogenesis. Neuron.

[72] Fernandez C.G., Hamby M.E., McReynolds M.L., Ray W.J. (2019). The Role of APOE4 in Disrupting the Homeostatic Functions of Astrocytes and Microglia in Aging and Alzheimer’s Disease. Front. Aging Neurosci..

[73] Weisgraber K.H., Innerarity T.L., Mahley R.W. (1982). Abnormal Lipoprotein Receptor-Binding Activity of the Human E Apoprotein Due to Cysteine-Arginine Interchange at a Single Site. J. Biol. Chem..

[74] Fagan A.M., Watson M., Parsadanian M., Bales K.R., Paul S.M., Holtzman D.M. (2002). Human and Murine ApoE Markedly Alters A Beta Metabolism before and after Plaque Formation in a Mouse Model of Alzheimer’s Disease. Neurobiol. Dis..

[75] Liao F., Zhang T.J., Jiang H., Lefton K.B., Robinson G.O., Vassar R., Sullivan P.M., Holtzman D.M. (2015). Murine versus Human Apolipoprotein E4: Differential Facilitation of and Co-Localization in Cerebral Amyloid Angiopathy and Amyloid Plaques in APP Transgenic Mouse Models. Acta Neuropathol. Commun..

[76] Tai L.M., Balu D., Avila-Munoz E., Abdullah L., Thomas R., Collins N., Valencia-Olvera A.C., LaDu M.J. (2017). EFAD Transgenic Mice as a Human APOE Relevant Preclinical Model of Alzheimer’s Disease. J. Lipid Res..

[77] Eisenberg D.T.A., Kuzawa C.W., Hayes M.G. (2010). Worldwide Allele Frequencies of the Human Apolipoprotein E Gene: Climate, Local Adaptations, and Evolutionary History. Am. J. Phys. Anthropol..

[78] Bekris L.M., Millard S.P., Galloway N.M., Vuletic S., Albers J.J., Li G., Galasko D.R., DeCarli C., Farlow M.R., Clark C.M. (2008). Multiple SNPs Within and Surrounding the Apolipoprotein E Gene Influence Cerebrospinal Fluid Apolipoprotein E Protein Levels. J. Alzheimers Dis. JAD.

[79] Husain M.A., Laurent B., Plourde M. (2021). APOE and Alzheimer’s Disease: From Lipid Transport to Physiopathology and Therapeutics. Front. Neurosci..

[80] Abondio P., Sazzini M., Garagnani P., Boattini A., Monti D., Franceschi C., Luiselli D., Giuliani C. (2019). The Genetic Variability of APOE in Different Human Populations and Its Implications for Longevity. Genes.

[81] Kern S., Mehlig K., Kern J., Zetterberg H., Thelle D., Skoog I., Lissner L., Blennow K., Börjesson-Hanson A. (2015). The Distribution of Apolipoprotein E Genotype over the Adult Lifespan and in Relation to Country of Birth. Am. J. Epidemiol..

[82] Kockx M., Traini M., Kritharides L. (2018). Cell-Specific Production, Secretion, and Function of Apolipoprotein E. J. Mol. Med. Berl. Ger..

[83] Morikawa M., Fryer J.D., Sullivan P.M., Christopher E.A., Wahrle S.E., DeMattos R.B., O’Dell M.A., Fagan A.M., Lashuel H.A., Walz T. (2005). Production and Characterization of Astrocyte-Derived Human Apolipoprotein E Isoforms from Immortalized Astrocytes and Their Interactions with Amyloid-Beta. Neurobiol. Dis..

[84] Bruinsma I.B., Wilhelmus M.M.M., Kox M., Veerhuis R., de Waal R.M.W., Verbeek M.M. (2010). Apolipoprotein E Protects Cultured Pericytes and Astrocytes from D-Abeta(1-40)-Mediated Cell Death. Brain Res..

[85] Xu Q., Bernardo A., Walker D., Kanegawa T., Mahley R.W., Huang Y. (2006). Profile and Regulation of Apolipoprotein E (ApoE) Expression in the CNS in Mice with Targeting of Green Fluorescent Protein Gene to the ApoE Locus. J. Neurosci. Off. J. Soc. Neurosci..

[86] Buttini M., Masliah E., Yu G.-Q., Palop J.J., Chang S., Bernardo A., Lin C., Wyss-Coray T., Huang Y., Mucke L. (2010). Cellular Source of Apolipoprotein E4 Determines Neuronal Susceptibility to Excitotoxic Injury in Transgenic Mice. Am. J. Pathol..

[87] Kockx M., Guo D.L., Huby T., Lesnik P., Kay J., Sabaretnam T., Jary E., Hill M., Gaus K., Chapman J. (2007). Secretion of Apolipoprotein E From Macrophages Occurs via a Protein Kinase A– and Calcium-Dependent Pathway Along the Microtubule Network. Circ. Res..

[88] Frenkel-Pinter M., Shmueli M.D., Raz C., Yanku M., Zilberzwige S., Gazit E., Segal D. (2017). Interplay between Protein Glycosylation Pathways in Alzheimer’s Disease. Sci. Adv..

[89] Boix C.P., Lopez-Font I., Cuchillo-Ibañez I., Sáez-Valero J. (2020). Amyloid Precursor Protein Glycosylation Is Altered in the Brain of Patients with Alzheimer’s Disease. Alzheimers Res. Ther..

[90] Haukedal H., Freude K.K. (2020). Implications of Glycosylation in Alzheimer’s Disease. Front. Neurosci..

[91] Flowers S.A., Grant O.C., Woods R.J., Rebeck G.W. (2020). O-Glycosylation on Cerebrospinal Fluid and Plasma Apolipoprotein E Differs in the Lipid-Binding Domain. Glycobiology.

[92] Schulman I.G. (2017). Liver X Receptors Link Lipid Metabolism and Inflammation. FEBS Lett..

[93] Spagnuolo M.S., Donizetti A., Iannotta L., Aliperti V., Cupidi C., Bruni A.C., Cigliano L. (2018). Brain-Derived Neurotrophic Factor Modulates Cholesterol Homeostasis and Apolipoprotein E Synthesis in Human Cell Models of Astrocytes and Neurons. J. Cell. Physiol..

[94] Zhao W., Fan J., Kulic I., Koh C., Clark A., Meuller J., Engkvist O., Barichievy S., Raynoschek C., Hicks R. (2020). Axl Receptor Tyrosine Kinase Is a Regulator of Apolipoprotein E. Mol. Brain.

[95] Hong C., Tontonoz P. (2014). Liver X Receptors in Lipid Metabolism: Opportunities for Drug Discovery. Nat. Rev. Drug Discov..

[96] Wood H. (2020). Retinoid X Receptor Mediates Brain Clean-up after Stroke. Nat. Rev. Neurol..

[97] Schierle S., Merk D. (2019). Therapeutic Modulation of Retinoid X Receptors—SAR and Therapeutic Potential of RXR Ligands and Recent Patents. Expert Opin. Ther. Pat..

[98] Fitz N.F., Nam K.N., Koldamova R., Lefterov I. (2019). Therapeutic Targeting of Nuclear Receptors, Liver X and Retinoid X Receptors, for Alzheimer’s Disease. Br. J. Pharmacol..

[99] Koldamova R., Lefterov I. (2007). Role of LXR and ABCA1 in the Pathogenesis of Alzheimer’s Disease—Implications for a New Therapeutic Approach. Curr. Alzheimer Res..

[100] Jacobo-Albavera L., Domínguez-Pérez M., Medina-Leyte D.J., González-Garrido A., Villarreal-Molina T. (2021). The Role of the ATP-Binding Cassette A1 (ABCA1) in Human Disease. Int. J. Mol. Sci..

[101] Fitz N.F., Cronican A.A., Saleem M., Fauq A.H., Chapman R., Lefterov I., Koldamova R. (2012). Abca1 Deficiency Affects Alzheimer’s Disease-Like Phenotype in Human ApoE4 But Not in ApoE3-Targeted Replacement Mice. J. Neurosci..

[102] Fernández-Calle R., Konings S.C., Frontiñán-Rubio J., García-Revilla J., Camprubí-Ferrer L., Svensson M., Martinson I., Boza-Serrano A., Venero J.L., Nielsen H.M. (2022). APOE in the Bullseye of Neurodegenerative Diseases: Impact of the APOE Genotype in Alzheimer’s Disease Pathology and Brain Diseases. Mol. Neurodegener..

[103] Mahley R.W. (2016). Central Nervous System Lipoproteins: ApoE and Regulation of Cholesterol Metabolism. Arterioscler. Thromb. Vasc. Biol..

[104] Nieweg K., Schaller H., Pfrieger F.W. (2009). Marked Differences in Cholesterol Synthesis between Neurons and Glial Cells from Postnatal Rats. J. Neurochem..

[105] Kim J., Yoon H., Basak J., Kim J. (2014). Apolipoprotein E in Synaptic Plasticity and Alzheimer’s Disease: Potential Cellular and Molecular Mechanisms. Mol. Cells.

[106] Lane-Donovan C., Wong W.M., Durakoglugil M.S., Wasser C.R., Jiang S., Xian X., Herz J. (2016). Genetic Restoration of Plasma ApoE Improves Cognition and Partially Restores Synaptic Defects in ApoE-Deficient Mice. J. Neurosci. Off. J. Soc. Neurosci..

[107] Tensaouti Y., Yu T.-S., Kernie S.G. (2020). Apolipoprotein E Regulates the Maturation of Injury-Induced Adult-Born Hippocampal Neurons Following Traumatic Brain Injury. PLoS ONE.

[108] Vitali C., Wellington C.L., Calabresi L. (2014). HDL and Cholesterol Handling in the Brain. Cardiovasc. Res..

[109] Hottman D.A., Chernick D., Cheng S., Wang Z., Li L. (2014). HDL and Cognition in Neurodegenerative Disorders. Neurobiol. Dis..

[110] Mahley R.W. (2016). Apolipoprotein E: From Cardiovascular Disease to Neurodegenerative Disorders. J. Mol. Med. Berl. Ger..

[111] Riddell D.R., Zhou H., Atchison K., Warwick H.K., Atkinson P.J., Jefferson J., Xu L., Aschmies S., Kirksey Y., Hu Y. (2008). Impact of Apolipoprotein E (ApoE) Polymorphism on Brain ApoE Levels. J. Neurosci. Off. J. Soc. Neurosci..

[112] Sullivan P.M., Han B., Liu F., Mace B.E., Ervin J.F., Wu S., Koger D., Paul S., Bales K.R. (2011). Reduced Levels of Human apoE4 Protein in an Animal Model of Cognitive Impairment. Neurobiol. Aging.

[113] Ramaswamy G., Xu Q., Huang Y., Weisgraber K.H. (2005). Effect of Domain Interaction on Apolipoprotein E Levels in Mouse Brain. J. Neurosci..

[114] Sullivan P.M., Mace B.E., Maeda N., Schmechel D.E. (2004). Marked Regional Differences of Brain Human Apolipoprotein e Expression in Targeted Replacement Mice. Neuroscience.

[115] Giannisis A., Patra K., Edlund A.K., Nieto L.A., Benedicto-Gras J., Moussaud S., de la Rosa A., Twohig D., Bengtsson T., Fu Y. (2022). Brain Integrity Is Altered by Hepatic APOE Ε4 in Humanized-Liver Mice. Mol. Psychiatry.

[116] Ulrich J.D., Burchett J.M., Restivo J.L., Schuler D.R., Verghese P.B., Mahan T.E., Landreth G.E., Castellano J.M., Jiang H., Cirrito J.R. (2013). In Vivo Measurement of Apolipoprotein E from the Brain Interstitial Fluid Using Microdialysis. Mol. Neurodegener..

[117] Linton M.F., Gish R., Hubl S.T., Bütler E., Esquivel C., Bry W.I., Boyles J.K., Wardell M.R., Young S.G. (1991). Phenotypes of Apolipoprotein B and Apolipoprotein E after Liver Transplantation. J. Clin. Investig..

[118] Getz G.S., Reardon C.A. (2009). Apoprotein E as a Lipid Transport and Signaling Protein in the Blood, Liver, and Artery Wall. J. Lipid Res..

[119] Huynh T.-P.V., Wang C., Tran A.C., Tabor G.T., Mahan T.E., Francis C.M., Finn M.B., Spellman R., Manis M., Tanzi R.E. (2019). Lack of Hepatic apoE Does Not Influence Early Aβ Deposition: Observations from a New APOE Knock-in Model. Mol. Neurodegener..

[120] Wahrle S.E., Shah A.R., Fagan A.M., Smemo S., Kauwe J.S.K., Grupe A., Hinrichs A., Mayo K., Jiang H., Thal L.J. (2007). Apolipoprotein E Levels in Cerebrospinal Fluid and the Effects of ABCA1 Polymorphisms. Mol. Neurodegener..

[121] Martínez-Morillo E., Hansson O., Atagi Y., Bu G., Minthon L., Diamandis E.P., Nielsen H.M. (2014). Total Apolipoprotein E Levels and Specific Isoform Composition in Cerebrospinal Fluid and Plasma from Alzheimer’s Disease Patients and Controls. Acta Neuropathol..

[122] Simon R., Girod M., Fonbonne C., Salvador A., Clément Y., Lantéri P., Amouyel P., Lambert J.C., Lemoine J. (2012). Total ApoE and ApoE4 Isoform Assays in an Alzheimer’s Disease Case-Control Study by Targeted Mass Spectrometry (N = 669): A Pilot Assay for Methionine-Containing Proteotypic Peptides. Mol. Cell. Proteomics MCP.

[123] Zokaei N., Giehl K., Sillence A., Neville M.J., Karpe F., Nobre A.C., Husain M. (2017). Sex and APOE: A Memory Advantage in Male APOE Ε4 Carriers in Midlife. Cortex J. Devoted Study Nerv. Syst. Behav..

